# Phenotypic heterogeneity drives differential disease outcome in a mouse model of triple negative breast cancer

**DOI:** 10.3389/fonc.2023.1230647

**Published:** 2023-09-29

**Authors:** Archana P. Thankamony, Sonny Ramkomuth, Shikha T. Ramesh, Reshma Murali, Priyanka Chakraborty, Nitheesh Karthikeyan, Binitha Anu Varghese, Vishnu Sunil Jaikumar, Mohit Kumar Jolly, Alexander Swarbrick, Radhika Nair

**Affiliations:** ^1^ Rajiv Gandhi Centre for Biotechnology, Trivandrum, Kerala, India; ^2^ Manipal Academy of Higher Education (MAHE), Manipal, Karnataka, India; ^3^ The Kinghorn Cancer Centre and Cancer Research Theme, Garvan Institute of Medical Research, Darlinghurst, NSW, Australia; ^4^ Centre for BioSystems Science and Engineering, Indian Institute of Science, Bangalore, India; ^5^ St Vincent’s Clinical School, Faculty of Medicine, UNSW Sydney, Darlinghurst, NSW, Australia; ^6^ Centre for Human Genetics, Bangalore, India

**Keywords:** intratumoral heterogeneity, TNBC, MACC1, phenotypic heterogeneity, Lovastatin

## Abstract

The triple negative breast cancer (TNBC) subtype is one of the most aggressive forms of breast cancer that has poor clinical outcome and is an unmet clinical challenge. Accumulating evidence suggests that intratumoral heterogeneity or the presence of phenotypically distinct cell populations within a tumor play a crucial role in chemoresistance, tumor progression and metastasis. An increased understanding of the molecular regulators of intratumoral heterogeneity is crucial to the development of effective therapeutic strategies in TNBC. To this end, we used an unbiased approach to identify a molecular mediator of intratumoral heterogeneity in breast cancer by isolating two tumor cell populations (T1 and T2) from the 4T1 TNBC model. Phenotypic characterization revealed that the cells are different in terms of their morphology, proliferation and self-renewal ability *in vitro* as well as primary tumor formation and metastatic potential *in vivo*. Bioinformatic analysis followed by Kaplan Meier survival analysis in TNBC patients identified Metastasis associated colon cancer 1 (Macc1) as one of the top candidate genes mediating the aggressive phenotype in the T1 tumor cells. The role of Macc1 in regulating the proliferative phenotype was validated and taken forward in a therapeutic context with Lovastatin, a small molecule transcriptional inhibitor of Macc1 to target the T1 cell population. This study increases our understanding of the molecular underpinnings of intratumoral heterogeneity in breast cancer that is critical to improve the treatment of women currently living with the highly aggressive TNBC subtype.

## 1 Introduction

Breast cancer is a highly heterogeneous disease and displays considerable diversity among patients (intertumoral heterogeneity) as well as within a tumor (intratumoral heterogeneity) (1, 2). The differences in the expression of hormonal receptors (ER/PR), human epidermal growth factor receptor 2 (HER2), Ki67 proliferation index and gene expression profiles among patients has enabled the classification of breast cancer into distinct subtypes: Luminal A, Luminal B, HER2 + and TNBC (1–4). The TNBC subtype constitutes 10-20% of all breast cancer cases (5–8) and is defined by the lack of expression of ER, PR and HER2 receptors (9–12). Consequently, patients with TNBC do not respond to hormonal or anti-HER2 therapies (13). Currently there are no molecular targeted therapies against TNBC and chemotherapy is the mainstay treatment option (8). Unfortunately, only 20% of TNBC cases respond to conventional chemotherapy (13) and is characterized by aggressive clinical course with cerebral and visceral metastasis. Compared to other subtypes, TNBC patients show significantly poor prognosis and lower overall survival (14, 15) making it imperative to devise new therapeutic strategies for targeting the disease.

Although TNBC is treated as a single clinical entity, recent genomic, transcriptomic and proteomic characterization has revealed profound heterogeneity with in TNBC (12, 16–20). Based on gene expression profiling, TNBC can be further classified into distinct molecular subtypes that have unique biological features and varied response to chemotherapy (21–24). Intratumoral heterogeneity refers to the presence of distinct cell populations within the same tumor with varying genetic, morphologic and phenotypic characteristics (25–28). This heterogeneity could arise as a consequence of cell intrinsic factors like genetic or epigenetic alterations, along with cell extrinsic or microenvironmental fluctuations (13). Intratumoral heterogeneity is regarded as a major driver of tumor progression, therapeutic resistance and metastasis (18, 29). Hence, increased understanding of the molecular mechanisms underlying this phenomenon is key for efficient therapeutic targeting of the TNBC subtype (30).

Accumulating evidences suggest that distinct cell populations derived from the triple negative breast cancer display varied tumor growth and metastatic potential (27, 29, 31). Various studies have shown that distinct tumor cell populations may also cooperate to promote tumor aggressiveness in breast cancer (31–33). For instance, Martin-Pardillos et al. isolated several clonal cell populations from the MDA-MB-231 TNBC cell line by marking different clones using fluorescent proteins (33). They found that some clones were able induce aggressiveness in other clones through soluble factors, extracellular vesicles or physical interactions. Another study by Kuiken et al. has shown that distinct clonal subpopulations derived from a human TNBC cell line MDA-MB-468 displayed significant functional heterogeneity and also varied in their growth dynamics under different contexts (34). These studies point to the importance of isolating heterogeneous tumor cell subpopulations from bulk cancers in order to unravel the molecular trappings underlying this phenomenon.

In order to dissect this further, we decided to investigate the 4T1 cell line, a widely used model system for studying TNBC heterogeneity. We were intrigued by an earlier study by Xiang et al. who observed two morphologically distinct tumor cell types following the *in vitro* culture of 4T1 primary tumor and lung metastases (35). One cell type displayed typical epithelial morphology while the other cell type was round and loosely touching the surrounding cells. However, these cells were not separately isolated or phenotypically characterized and the contribution of these two morphologically distinct tumor cells towards disease outcome was also not studied thoroughly. We have now successfully isolated two distinct tumor cell types from the primary tumor of the 4T1 model system (T1 and T2) which exhibited different *in vitro* phenotypes as well as *in vivo* disease outcomes (36). We further employed an unbiased transcriptomic analysis of the two cell populations in order to identify the key mediator of this phenotypic heterogeneity, thus opening up exciting new avenues to explore targeting of aggressive subpopulations of cells within a tumor.

## 2 Materials and methods

### 2.1 Cell culture

Mouse breast cancer cell line, 4T1, stably expressing green fluorescent protein (4T1GFP) was cultured in RPMI 1640 (11875119, Thermo Fischer Scientific) supplemented with 10% FBS (10270106, Thermo Fischer Scientific), Penicillin (100 Units/mL)/Streptomycin (100µg/mL) (15070063, Thermo Fischer Scientific), 0.25% (v/v) D+ Glucose (G8644, Sigma Aldrich), 1mM sodium pyruvate and 20mM HEPES. The primary cells were cultured in DMEM/F-12 (11320082, Thermo Fisher Scientific) supplemented with 10% FBS and Penicillin (100 Units/mL)/Streptomycin (100µg/mL). All the cells were grown in 5% CO2 incubator at 37°C.

### 2.2 *In vivo* 4T1GFP tumor model

All animal experiments were conducted in accordance with RGCB institutional animal ethics committee. In order to generate 4T1GFP spontaneous metastasis model 7x10^3^ 4T1GFP cells were injected into the fourth mammary fat pad of six to eight weeks old BALB/c mice. For the resection experiment, the primary tumor was surgically removed when the tumor reached 0.8 cm^3^ volume. For experimental metastasis assay, 5x10^5^ T1 and T2 cells in 100 µL DPBS were injected via tail vein (n=5). At ethical end point (as indicated by reduction in body weight and acute respiratory stress), mice were sacrificed and lung metastases were harvested. For long term survival analysis, mice were orthotopically injected with 7x10^3^ T1 and T2 cells (n=5 and n=9 respectively). Survival analysis was performed using GraphPad Prism software.

### 2.3 Tumor dissociation and flow cytometric analysis of cell populations

Tumors harvested from the mice were dissociated into single cell suspension for FACS sorting. Tumor dissociation into single cell suspension was carried out using the MACS mouse Tissue Dissociation Kit (Miltenyi Biotec, USA) using manufacturer’s protocols. Following the dissociation 1x10^6^ tumor cells were washed once with DPBS with salts. The cells were then incubated in FcR blocking reagent (130-092-575, Miltenyi Biotec, USA) for 10 minutes on ice. After incubation, the cells were washed twice using DPBS with salts. Cells were stained for 20 minutes on ice with fluorochrome conjugated antibodies against lineage markers EpCam-FITC (130-102-995, Miltenyi Biotec, USA), CD140a-PE (130-102-502, Miltenyi Biotec, USA). Prior to analysis the cells were washed twice and resuspended in FACS buffer (DPBS with salts + 20% FBS + 20% HEPES). The stained tumor cells were analyzed using the flow cytometer (BD FACS Aria III, BD Biosciences, San Jose, CA, USA).

### 2.4 Cell proliferation assay

5x10^2^ T1 and T2 tumor cells were seeded into 96 well plates and the cell proliferation were measured for 72 hours using MTT (3-(4,5-dimethylthiazol-2-yl)-2,5-diphenyltetrazolium bromide, M6494, Thermo Fischer Scientific) assay. Briefly, 10 µl MTT solution (5mg/ml) was added to each well and incubated for 2 hours at 37°C. After incubation, the plates were centrifuged at 1200 rpm for 5 minutes at room temperature and the media was removed. The purple-colored formazan crystals were dissolved by adding 100 µL DMSO (D5879, Sigma Aldrich) and the absorbance at 570 nm was measured using plate reader (Tecan)

### 2.5 Tumorsphere assay

For sphere assay, T1 and T2 cells were trypsinized and washed with DPBS. These cell populations were then resuspended in serum free DMEM/F-12 media supplemented with 1X B27 (17504-044, Thermo Fischer Scientific), 20ng/mL bFGF (GF003, Sigma Aldrich), 4µg/mL Heparin (H3149-50KU, Sigma Aldrich). Both the cells were seeded at a density of 1x10^3^ cells per well in an ultra-low attachment 24 well plate (Corning, Sigma). Media was replenished every third day and the tumorspheres were counted by day 5.

### 2.6 Quantitative real time PCR (qRT-PCR)

Total RNA was isolated using Trizol^®^ reagent (15596026, Thermo Fischer Scientific) and 1.5 µg of RNA was used for cDNA preparation using the High-Capacity cDNA Reverse Transcription Kit (4374966, Thermo Fisher Scientific). Real-time PCR was performed using iTaq Univer SYBR Green (1725121, BioRad) on the QuantStudio 7 Flex RealTime PCR System (Applied Biosystems). The PCR conditions are 95° C for 10 minutes, followed by 40 cycles of 95° C for 30 seconds, and 60° C for 1 minute. All reactions were performed in triplicates and the transcript levels are normalized to those of β-actin. The relative fold change is determined by 2^−ΔΔCT^ method.

### 2.7 Hematoxylin & Eosin (H & E) staining

Breast tumor tissues from mice were placed on the sample cassettes and fixed in 10% neutral buffered Formalin (24-72 hours) and transferred into PBS. Samples were embedded in paraffin and 5µm thick sections were taken. Subsequently, the tissue sections were stained using Hematoxylin and Eosin (H &E) and examined for metastases. After incubating at 60° C for one hour, the sections were deparaffinized in Xylene followed by rehydration in graded isopropanol solutions. The slides were then rinsed in tap water and kept in acid alcohol for 3 seconds again followed by tap water wash. Subsequently, the slides were dipped in bluing solution three-four times. Afterwards, the slides were rinsed thrice with tap water and transferred into 70% isopropanol for 3 minutes. Following this, the sections were stained with Eosin Y for 10 minutes. After staining, dehydration was performed in graded isopropanol followed by clearing in Xylene. The slides were then mounted with DPX and were visualized using bright field microscope (Leica).

### 2.8 RNA sequencing

Total RNA from three replicates each of 4T1GFP (parental cell line), T1 and T2 cells were isolated using the RNeasy minikit (74104, Qiagen) according to manufacturer’s instructions. RNA concentration of the samples was estimated using Qubit RNA Assay BR (Q10211, Invitrogen). RNA purity was determined using QIAxpert and RNA integrity was measured using RNA ScreenTapes (5067-5576, Agilent) on TapeStation. All the 9 RNA samples passed the QC and were used for the library prep. A modified Illumina TruSeq RNA Sample Preparation V2 protocol (RS1222001) was used for making RNA sequencing libraries. The initial RNA Concentration of 500 ng was taken. First, the total RNA sample was subjected to mRNA enrichment by using Poly(A) purification beads. After purification, the mRNA was fragmented under elevated temperatures using divalent cations followed by cDNA synthesis. Indexed adapters were ligated to the cDNA and was then purified and enriched using the following thermal conditions: initial denaturation 98°C for 30 seconds; 13 cycles of 98°C for 10 seconds, 60°C for 30 seconds, 72°C for 30 seconds; final extension of 72°C for 5minutes. PCR products were then purified and checked on TapeStation D1000 DNA tapes (5067-5582, Agilent) for fragment size distribution. Using Qubit High Sensitivity Assay (Q32852, Invitrogen), the prepared libraries were quantified. Before cluster amplification, the libraries were pooled and diluted to the final loading concentration. After completing the cluster generation, the flow cell was loaded on Illumina HiSeqX instrument and 150bp paired end reads were generated.

### 2.9 Bioinformatic analysis

The FASTq files obtained after sequencing was checked for quality using FASTQC software. Following the quality check, the adapters were removed from the sequences using Trimmomatic tool. After trimming the adaptors and subsequent quality check, the reads were aligned to the mouse reference genome (Mm10) using TopHat2. HTSeq tool was used to obtain the read counts after alignment. The obtained read counts were normalized and differential gene expression analysis was performed using DESeq2. The genes which showed a false discovery rate (FDR) adjusted p value (q value) < 0.01 and Log2FC >2 were considered statistically significant. Heatmap of the differentially ex-pressed genes were constructed using HeatMap tool in the GenePattern software.

### 2.10 Kaplan Meier plotter

The top 50 differentially regulated genes with q value < 0.01 and Log2FC >2 were filtered based on their prognostic significance in triple negative breast cancer (TNBC) patients. The association of candidate gene expression with the survival of TNBC patients were analyzed using Kaplan-Meier plotter (KM plotter) database (http://kmplot.com/analysis/) which derives the gene expression and survival data of breast cancer patients from the gene expression omnibus (GEO) database. After entering the gene list in to the KM plotter database, the patient samples were split by auto-selecting best cut-off. The association of relapse free survival (RFS, n=152) and distant metastasis free survival (DMFS, n=137) with each candidate gene was determined. The analysis was performed on breast cancer patients with negative ER, PR and HER2 status). The hazard ratio (with confidence intervals) and p values were obtained from the KM plotter.

### 2.11 Analysis of single cell transcriptomic data of human breast cancer samples

The human breast cancer single-cell RNA-seq dataset previously reported by Wu et al. (37) was downloaded from the Broad Institutes Single Cell Portal (https://singlecell.broadinstitute.org/single_cell/study/SCP1039/a-single-cell-and-spatially-resolved-atlas-of-human-breast-cancers#study-download). For further information detailing pre-processing, clustering, and cell type annotation, please refer to the authors publication. The data was loaded into R and recompiled into a Seurat (v4) (38) object using the sourced matrix, feature, and barcode files. The Seurat object was sub-set to include only barcodes identified as “cancer epithelial” using the metadata provided by the authors; similarly, the UMAP embedding was recreated using UMAP coordinates obtained from the single cell portal. Only the TNBC clusters were used for further analysis. The TNBC clusters were broadly stratified into three groups (low, med, high) based on the average expression of MACC1 and visualized using plotting methods implemented in various R packages. Since the authors reported cluster segregation being strongly driven by patient of origin, this binning procedure was also applied to cluster 4 in isolation. Cluster 4 was chosen due to high relative intratumoral heterogeneity alongside cells with high MACC1 expression. The statistical analyses described were executeded on both the aggregated TNBC clusters and on cluster 4 alone, however, to minimize patient specific differences unrelated to MACC1 expression and ensure robust comparisons, a focus was put on cluster 4. Differentially expressed genes (DEGs) were identified using the MAST (39) statistical framework implemented in Seurat’s ‘FindMarkers’ method. The analysis was conducted on the high vs. low MACC1-expressing subpopulations of cluster 4. DEGs with an average log 2 fold change >= 0.5 and an adjusted p-value <0.05 were used as input to the R package “enrichR”, which provides an interface to the Enrichr database (40) A hypergeometric test was performed using enrichR to determine enrichment of Gene Ontology terms associated with the “GO_Biological_Process_2021”, “GO_Molecular_Function_2021”, and “GO_Cellular_Component_2021” databases (40, 41) Pathway enrichment was also evaluated using “The Kyoto Encyclopedia of Genes and Genomes” (KEGG) database (41). To assess gene co-expression, pairwise Pearson correlations were computed between MACC1 and every other expressed gene in cluster 4 using the ‘cor.test’ function implemented by the “Stats” R package. To counteract the impact of multiple comparisons, a Bonferroni correction was applied to each p-value. The same methods were repeated post removal of ribosomal genes.

### 2.12 siRNA mediated knockdown of Macc1

6x10^3^ T1 cells were seeded in 96 well plates. For small interference RNA (siRNA) mediated knockdown of Macc1, T1 cells were reverse transfected with 50 nM of universal negative control (control) and targeting MISSION^®^ Predesigned siRNA (Sigma Aldrich) using Lipofectamine 2000 following manufacturer’s instructions in optiMEM (31985062, Themo Fisher Scientific) media. After 24 hours, media was changed with fresh DMEM/F-12 media. The cell proliferation 48 hours post transfection was analyzed by MTT assay. For RNA isolation, 2.5x10^5^ T1 cells were seeded in a 6 well plate. 48 hours after transfection, RNA was isolated from the plates using Trizol method.

### 2.13 Lovastatin treatment

Lovastatin was obtained from Santacruz biology and was dissolved in dimethyl sulfoxide (DMSO). 10 mM stock of Lovastatin was stored at -20° C in aliquots to avoid repeated freeze thawing. To see the effect of Lovastatin on cell proliferation, 1x10^3^ T1 cells were seeded in 96 well plate. After 24 hours, the cells were treated with increasing concentrations of Lovastatin (1, 2.5, 5, 7.5 and 10 µM). T1 cells treated with an equal amount of solvent (DMSO) was used as the control. The cell proliferation 48 hours post Lovastatin treatment was analyzed by MTT assay. For RNA isolation, 5x10^5^ T1 cells were seeded in 60 mm cell culture plates. After 24 hours, the cells were treated with 6 µM Lovastatin. Total RNA was isolated from Control and Lovastatin treated cells using Trizol method 48 hours post Lovastatin treatment.

### 2.14 Western blotting

T1 and T2 cells were lysed and protein was isolated using Radioimmunoprecipitation assay (RIPA) lysis buffer (R0278-50ML, Sigma-Aldrich) with protease inhibitor (P8340-5ML, Sigma-Aldrich). The protein concentrations were quantified using BCA assay (23225, Thermo Fisher Scientific). 25 µg of total protein lysate was loaded on to 10% SDS gel and western blotting was performed as previously described (42). Antibodies used in the study are listed in **Supplementary Table 5**.

### 2.15 Statistical analysis

Statistical analyses were performed using GraphPad Prism 6 (GraphPad Software, Inc., San Diego, CA, USA). All experiments were performed in at least three biological replicates. Data are represented as mean ± standard deviation. Statistical tests used are un-paired Student’s t-test and two-way ANOVA. p-values < 0.05 were considered statistically significant with * p < 0.05, ** p < 0.01, *** p < 0.001, **** p < 0.0001.

## 3 Results

### 3.1 Two distinct tumor cell populations were obtained from the primary tumor

**Figure 1:**
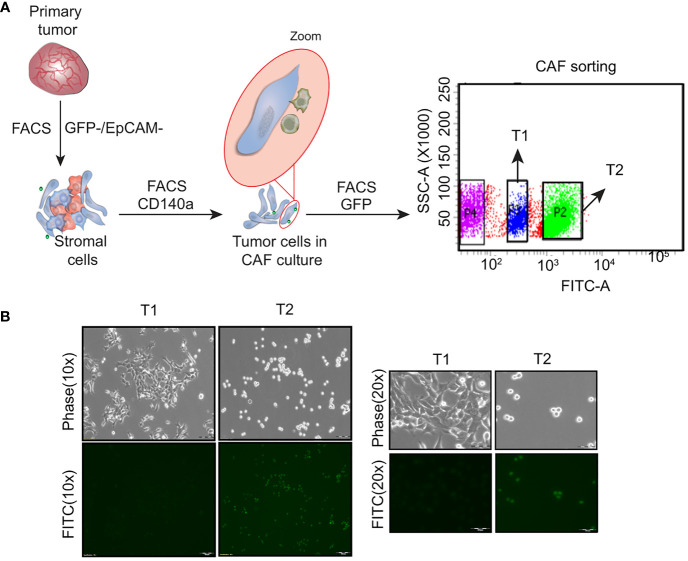
Isolation of heterogeneous tumor cell populations from primary tumor **(A)** Schematic showing the dissociation and sorting of primary tumor based on the expression of GFP and EpCam. Two distinct tumor cell populations were obtained following the sorting - an adherent cell population named as T1 and a round cell population named as T2 **(B)** Representative phase contrast and fluorescent microscopy images showing the T1 and T2 tumor cell populations. (Magnification 10x, 20x).

Heterogeneous tumor cell populations were isolated from the 4T1GFP syngeneic tumor model as shown in **Supplementary Figure 1**. The primary tumor was surgically resected 30 days post injection (when the tumor reached the ethical volume) to recapitulate the resection that patients undergo in a clinical setting The primary tumor was then dissociated into single cells and sorted by flow cytometry into tumor epithelial (GFP+) and stromal fractions (GFP-) (**Figure 1A**; **Supplementary Figure 2A**). The stromal population was further sorted based on the expression of CD140a to obtain cancer associated fibroblasts (CAFs) (GFP-/CD140a+) (**Supplementary Figure 2B**). However, we noticed the presence of few GFP+ tumor epithelial cells along with the fibroblast cells when the sorted CAF (GFP-) cells were cultured. As shown in **Supplementary Figure 2C**, the GFP+ tumor epithelial cells eventually outnumbered the CAFs. We then resorted this CAF-tumor cell mixed population based on GFP expression to separate the GFP+ tumor epithelial cells (**Figure 1A**). Two tumor epithelial cell populations that showed distinct GFP expression levels were obtained from the GFP+ fraction - a GFP^High^ (High GFP expression) and a GFP^Low^ (Low GFP expression) cell population. Interestingly, these cell populations also displayed different morphological appearance when cultured *in vitro* which was similar to observations made by Xiang and colleagues (**Figure 1B**) (14). The GFP^High^ cells showed a round and loosely adherent morphology whereas GFP^Low^ cell population exhibited adherent, 4T1 like morphology. These cells will be referred to as T1 (GFP^Low^, Adherent) and T2 (GFP^High^, Round) in the following sections.

### 3.2 *In vitro* characterization of T1 and T2 tumor cell populations

**Figure 2:**
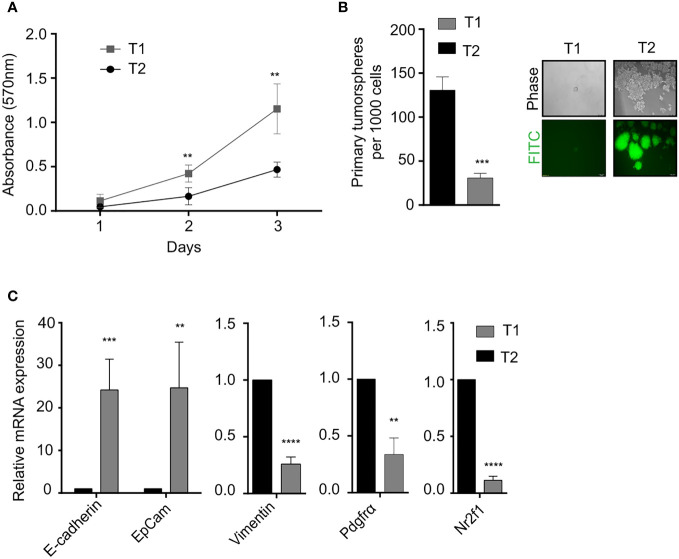
Phenotypic characterization of T1 and T2 tumor populations from primary tumor **(A)** Proliferation analysis using MTT assay shows that T1 cells proliferated at a significantly faster rate. **(B)** Quantification of the number of primary tumorspheres formed by the T1 and T2 populations in tumor-sphere assay. Phase contrast and fluorescence microscopy images showed that T1 cells formed fewer and smaller tumorspheres **(C)** Quantitative real-time PCR showing the relative mRNA expression of epithelial-mesenchymal and quiescence genes in the T1 and T2 populations from the primary tumor. Data are expressed as mean ± standard deviation. n=3, p <0.05 are considered statistically significant with **p<0.01, ***p<0.001, ****p<0.0001.

*In vitro* phenotypic characterization of the T1 and T2 tumor cell populations was performed to study the observed morphological heterogeneity at a functional level. Proliferation analysis using the MTT assay revealed that the T1 cells showed significantly higher proliferation rate compared to T2 cells (**Figure 2A**). We next looked at a key attribute of cancer cells, self-renewal ability and observed that the T2 cells showed significantly higher self-renewal ability compared to T1 cells as indicated by the number of primary tumorspheres (**Figure 2B**). The T1 and T2 tumor cells were also different in terms of the expression of epithelial markers (EpCam, E-Cadherin), mesenchymal markers (Vimentin, Pdgfra) and dormancy associated marker (Nr2f1) as shown by the qRT-PCR analysis (**Figure 2C**).

### 3.3 Phenotypic heterogeneity is linked to distinct disease outcome

**Figure 3:**
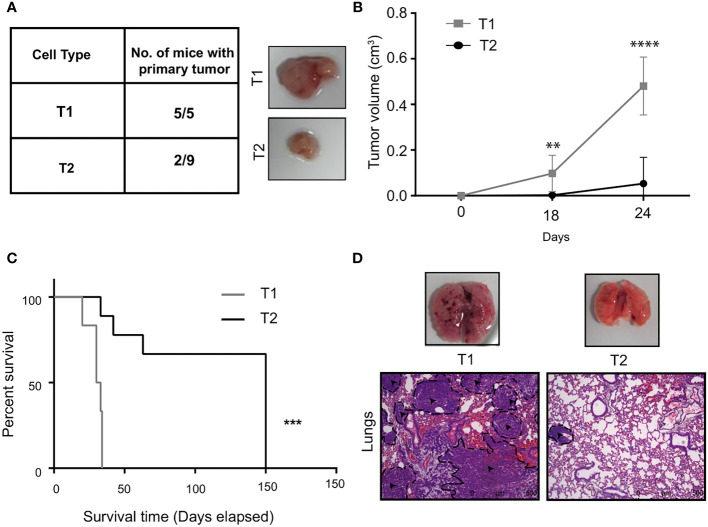
T1 cells showed highly aggressive disease outcome **(A)** *In vivo* tumorigenic potential of T1 and T2 tumor cells were analyzed by injecting 7x10^3^ tumor cells orthotopically into the fourth mammary fat-pad of BALB/c mice. Table shows the number of mice that developed primary tumors/number of mice injected. Representative image shows the primary tumors formed by T1 and T2 tumor cells after 4 weeks. The T1 cells are highly aggressive as they formed larger primary tumors compared to the T2 cells **(B)** Tumor growth curves of mice injected with T1 (n=5) and T2 cells (n=9) **(C)** Percentage survival of mice injected with T1 and T2 tumor cells. **(D)** Representative H&E staining of lungs of mice injected with 5x10^5^ T1 and T2 tumor cells via the lateral tail veins (n=5). The black dashed lines and arrows indicating the metastatic lesions. Magnification 10x. **p<0.01, ***p<0.001, ****p<0.0001.

The results from the *in vitro* characterization showed that we had successfully isolated two distinct tumor cells from the bulk cancer mass. We next addressed the question if the *in vitro* phenotypic heterogeneity we observed, translated to different tumorigenic capacity *in vivo*. The T1 and T2 tumor cells were orthotopically injected into the mammary fat-pad of 6 to 8 week old BALB/c female mice and primary tumor growth was monitored over 30 days. Interestingly, all the mice (5/5) that were injected with the T1 tumor cells formed primary tumors. In contrast, only 2 out of the 9 mice injected with the T2 cells formed tumors that were significantly smaller than the T1 tumors (**Figures 3A, B**). This fits in well with our observation that T1 tumor cells showed higher proliferation rate *in vitro* when compared to T2 cells. We next performed the Kaplan Meier survival analysis on the mice injected with T1 and T2 tumor cells (**Figure 3C**). Mice injected with the T1 tumor cells showed a median survival of 31 days whereas the mice injected with T2 cells showed a median survival of 150 days. Log-rank test revealed that mice injected with T1 tumor cells had significantly lower survival rate compared to mice injected with the T2 tumor cells (p = 0.0004). We also analyzed the metastatic potential of these two cell types using the experimental metastasis assay. We injected the T1 and T2 tumor cells via the lateral tail vein of BALB/c mice (n=5 each). After sacrificing the mice, lungs were harvested and examined for metastases. As shown by the representative H&E staining, all the mice injected with the T1 cells formed large lung macrometastases (5/5) (**Figure 3D**). The mice injected with the T2 cells did not exhibit lung metastasis in all the samples (4/5) and the metastases formed were smaller in size (average number of metastases per mouse = 6.4, **Supplementary Table 1**). The highly proliferative T1 cells formed large macrometastases in all the mice (5/5) and could not be quantified. The *in vivo* results clearly showed that the T1 tumor cells have significantly higher tumorigenicity and metastatic potential compared to the T2 cells (**Supplementary Table 2**).

### 3.4 Identification of candidate genes associated with poor prognosis of TNBC patients

To identify the differentially expressed genes involved in mediating the phenotypic het-erogeneity in the 4T1 primary tumor and aggressive phenotype of T1 cells compared to the T2 cells, we performed transcriptomic analysis on the 4T1GFP (parental), T1 and T2 cells using RNA sequencing (**Supplementary Figure 3**). RNA sequencing was performed using the Illumina HiSeqX platform. The read counts were normalized and differential gene expression analysis was performed using DESeq2 which uses median of ratios as normalization method. The top candidate genes were then identified based on the filters such as q-value < 0.01 and Log2 fold change >2 (**Supplementary Figure 3**). Based on the filters, top 50 candidate genes upregulated in the T1 compared to T2 population were obtained (**Supplementary Table 3**).

**Figure 4:**
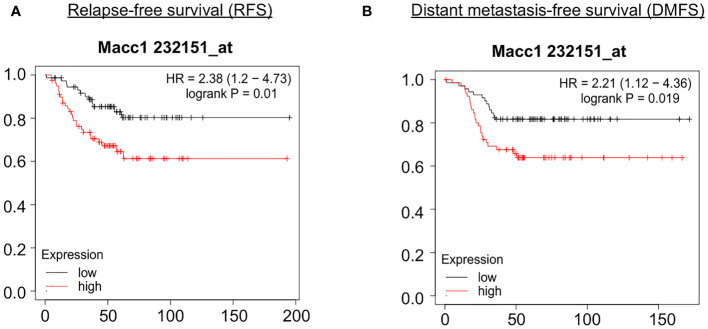
Association of relapse and distant metastasis-free survival of TNBC patients with expression of MACC1 Correlation of MACC1 gene expression with TNBC patient survival was analyzed using KM plotter. TNBC patients with higher expression (red line) of MACC1 was associated with significant lower relapse-free survival **(A)** and distant metastasis-free survival **(B)** compared to the patients with lower expression of MACC1 (black line).

In order to identify the genes contributing to the aggressive disease phenotype in the T1 cell population, we analyzed the association of the top 50 candidate gene’s expression with relapse and distant metastasis free survival (RFS and DMFS) in breast cancer using publicly available curated clinical datasets like TGCA in the Kaplan Meier (KM plotter) software. Macc1 was identified as the top gene for further validation as TNBC patients with a higher mean expression of MACC1 showed poor relapse-free survival (RFS - Hazards ratio/HR: 2.38, log p value: 0.01) and distant metastasis-free survival (DMFS, HR: 2.21, log p value: 0.019) compared to the patients with lower MACC1 expression (**Figures 4A, B**). We have analyzed the expression of Macc1 at protein level in the T1 and T2 cells. T1 cells showed significantly higher expression of Macc1 compared to T2 cells (**Supplementary Figure 5B**) validating our RNA sequencing analysis.

### 3.5 Investigating the correlation of higher MACC1 expression with aggressive disease phenotype in human TNBC patients at single cell level

**Figure 5:**
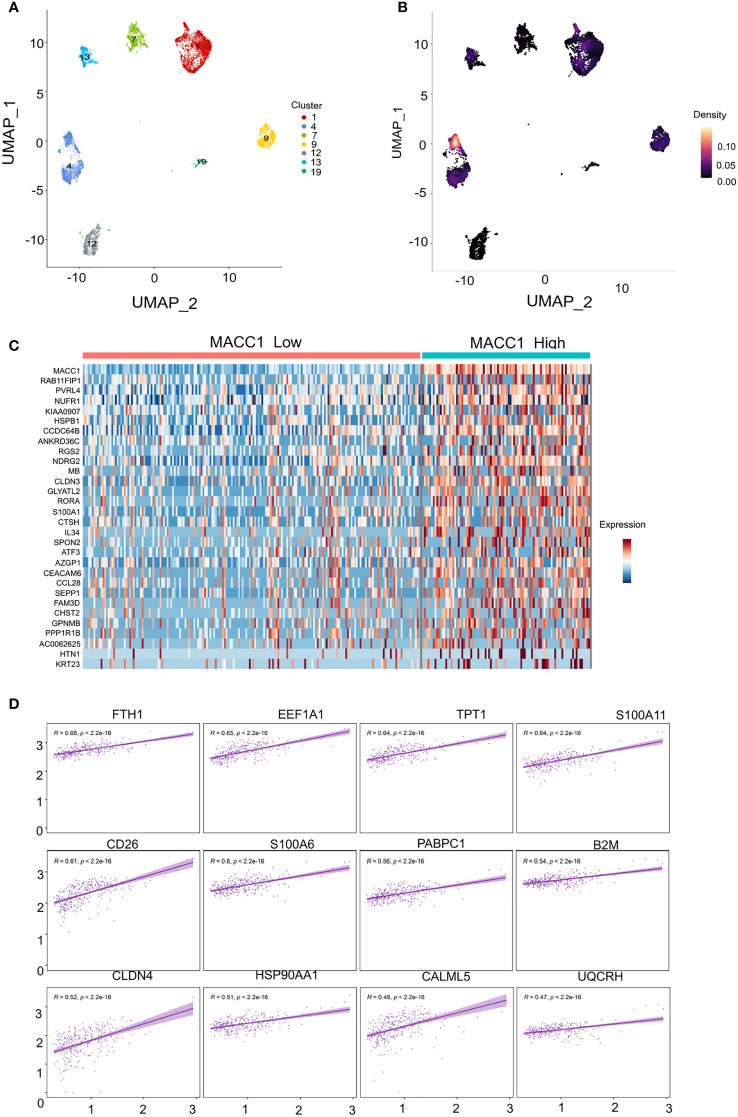
Single cell transcriptomic analysis of MACC1 expression in human TNBC samples **(A)** UMAP visualization of all malignant epithelial cells in the human TNBC samples (n= 7) **(B)** Density UMAP visualization of MACC1 expression in each of the TNBC samples (Yellow color showing highest MACC1 expression and black showing the least. **(C)** Heatmap showing top 15 up and down regulated genes associated with MACC1 high versus low cells **(D)** Top 12 positively correlated genes associated with MACC1 expression in cluster 4 (after excluding the ribosomal genes).

We next took the important step of correlating differential MACC1 expression with disease phenotype in human patient samples. We analyzed previously published single cell RNA sequencing data (37) and stratified the malignant epithelial cells based on molecular subtypes. We only retained the TNBC annotated clusters using the UMAP coordinates which resulted in 7 clusters with each cluster representing a single tumor/patient sample (**Figure 5A**). We screened each TNBC cluster for MACC1 expression using gene-weighted density estimation based on the log normalized counts of each cell (**Figure 5B**). In order to minimize potential noise from patient-specific differences, we focused on only one cluster - cluster 4, as it showed highest overall Macc1 expression. Interestingly, cluster 4 also displayed higher intratumoral heterogeneity within the malignant individual cells leading to a clear bifurcation in the cluster (**Figure 5B**). In order to investigate the intratumoral heterogeneity in the MACC1 expression in cluster 4 (**Figure 5C**), we binned the cells based on their average MACC1 expression. We collapsed the medium and high expression groups into a single group to improve the power of our comparisons though it reduced the magnitude of the average expression value of MACC1 between the groups being compared (low MACC1 expressing cells = 189 and medium/high MACC1 expressing cells = 94). Interestingly, a clear distinction in the gene expression pattern of cluster 4 was observed (**Figure 5C**). To identify the genes and pathways associated with higher MACC1 expression, we looked at the genes that are differentially regulated in the MACC1 high versus low cells in cluster 4 (**Figure 5C**). Cells assigned to the high MACC1 expression bin were found to be enriched in genes associated with the gene ontology (GO) term ‘positive regulation of angiogenesis’(data not shown). This is interesting as MACC1 has already been reported to promote angiogenesis in many cancers (43). In addition, we also performed the analysis of the pair wise gene correlation based on Pearson’s correlation coefficient and identified the top 12 genes that showed significant positive correlation with MACC1 expression (**Figure 5D**). The functional relevance of these genes in breast cancer progression was then determined. Among the positively correlated genes, EF1A1, TPT1, S100A11, PABPC1, B2M, CLDN4, HSP90AA1, UQCRH were found to be associated with aggressive disease phenotype in breast cancer (44–50). For example, EF1A1 (Eukaryotic translation elongation factors 1 alpha 1), is known to promote tumor cell proliferation, migration and invasion in TNBC cells. Our results point to the molecular mechanism by which MACC1 can contribute to the aggressive disease phenotype in human TNBC which is being explored further in future work.

### 3.6 Macc1 depletion caused significant reduction in breast cancer cell proliferation

**Figure 6:**
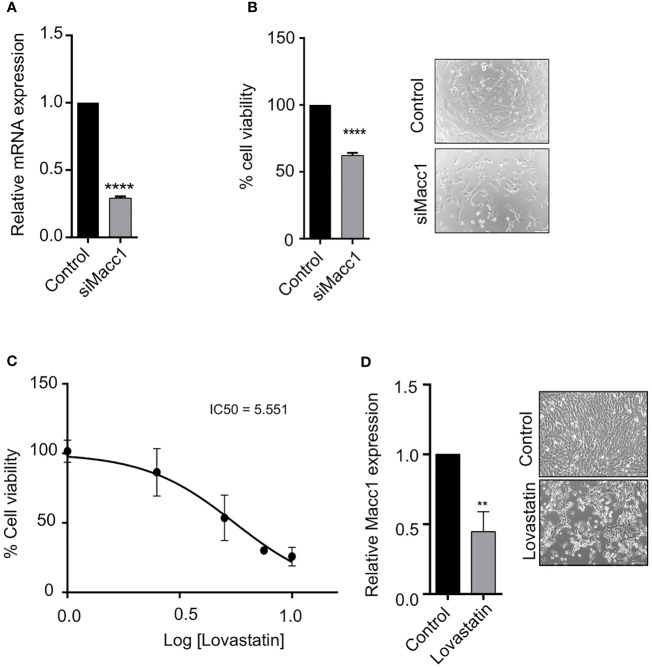
Targeting Macc1 expression affected the proliferation of aggressive breast cancer cells **(A)** Relative expression of Macc1 mRNA in T1 cells transfected with Macc1 siRNA compared to the control as quantified by the qRT-PCR. **(B)** Percentage cell viability of T1 cells following transfection of control and Macc1 siRNAs. The cells were reverse transfected with 50nM siRNAs and the cell proliferation after 48 hours were analyzed using MTT assay. Knockdown of Macc1 caused a significant reduction in the percentage cell viability compared to control. Phase contrast microscopy images showing the T1 cells transfected with Macc1 siRNA compared to control cells are shown as inset. **(C)** Graph showing the percentage cell viability of T1 cells after Lovastatin treatment. T1 cells were treated with different concentrations of Lovastatin (1, 2.5, 5, 7.5 and 10 µM) and cell viability after 48 hours was measured using MTT assay. The IC50 concentration of Lovastatin for T1 cells was then calculated using GraphPad Prism software. **(D)** The relative mRNA expression of Macc1 in Lovastatin (6 µM) treated cells compared to control as analyzed by qRT-PCR. Macc1 expression was normalized to β-actin. Phase contrast microscopy images showing the T1 cells treated with Lovastatin (6 µM) compared to DMSO treated (control) cells are shown as inset. n=3, p <0.05 are considered statistically significant with **p<0.01, ****p<0.0001.

Macc1 is known to regulate a number of cellular functions in cancer including cell proliferation. We performed siRNA-based silencing of Macc1 in the aggressive T1 cells in order to evaluate the role of Macc1 gene expression in breast cancer cell proliferation. We first looked at the mRNA levels to determine if the knock down had worked. A significant reduction in the expression of Macc1 was observed 48 hours post-transfection (**Figure 6A**). Interestingly, Macc1 silencing resulted in significantly lower cell viability (**Figure 6B**) in the knock down tumor cells (siMacc1) compared to T1 tumor cells (Control).

We were interested in extending this finding to a clinical context by using Lovastatin, a small molecule transcriptional inhibitor of Macc1 (51, 52). T1 cells were treated with increasing concentrations of Lovastatin for 48 hours (**Figure 6C**). Treatment with Lovastatin resulted in a significant decrease in the T1 cell proliferation in a dose dependent manner (**Figure 6C**). We further analyzed the effect of Lovastatin on Macc1 expression. The expression of Macc1 mRNA was significantly diminished after Lovastatin treatment (**Figure 6D**). This result demonstrates proof of principle that we can target the T1 aggressive cells within a heterogenous tumor mass effectively via Macc1, by using the small molecule inhibitor Lovastatin.

## 4 Discussion

Breast cancer manifests itself in different clinico-pathological forms depending on underlying gene signatures. Intertumoral heterogeneity has been at the heart of treatment modalities of breast cancer with the success in breast cancer management arising largely from the use of targeted therapy like Tamoxifen in ER+ and Herceptin for the Her2 subtypes. But the increasing spotlight on the different cell types within a single tumor termed intratumoral heterogeneity results in the challenging situation where drug resistant cells escape and lead to relapse of the tumor. This is particularly true in the TNBC molecular subtype which though classified as a single entity based on immuno-histochemistry (IHC), actually is a group of diseases. More recent work has shown that there could also be unique “ecotypes” within tumors involving the interaction of cancer cells with the microenvironment (37) thus complicating the picture. Our work aims to deconvolute the different cell populations within a single TNBC tumor and understand if we could exploit the underlying molecular circuitry for therapeutic purposes.

The 4T1 model had previously been reported to have morphologically distinct cell types by Xiang et al., although they did not isolate and study the relationship of morphologically distinct tumor cells with disease outcomes due to technical challenges (35). We overcame this hurdle by tagging the heterogenous bulk cell population with a GFP tag, which enabled us to isolate multiple tumor cells based on their GFP expression and culture them *in vitro*. We decided to focus our current work on two cell types that displayed striking morphological distinctness in multiple passages *in vitro*. More importantly, the morphological difference also translated into differing growth kinetics, self-renewal capacity, EMT molecular markers and contrasting disease outcomes *in vivo*. This confirmed our hypothesis that a tumor is composed of non-identical cells which coexist to give different functionality to the cancer (33). We hypothesized that the slow cycling T2 cells are more stem-like when compared to T1 cells. We observed a significantly higher self-renewal ability in T2 cells in the tumorsphere assay (**Figure 2B**). In addition, T2 cells have higher expression of a dormancy associated marker Nr2f1 (4, 5) (**Figure 2C**) pointing towards their quiescent nature. In order to further test the *in vivo* self -renewal and tumor propagating capacity of the T1 and T2 cells, we will perform *in vivo* long-term tumor growth assays as a part of our future study.

In addition to this, we analyzed the EMT scores of the three cell populations by applying three different scoring methods – a 76 gene EMT signature (76GS), Kolmogorov Smirnov (KS) method and Multinomial logistic regression (MLR) method (Chakraborty et al., 2020) using the global gene expression data, to determine the epithelial-mesenchymal status of the cells. Interestingly, the EMT score calculation by all the three methods showed that the T2 cells are more mesenchymal than the 4T1GFP parental and T1 cells which aligns with our *in vitro* findings (**Supplementary Figure 4**, **Supplementary Table 4**). We have also analyzed the expression of E-Cadherin and Vimentin at protein level. We observed a high expression of E-Cadherin in the T1 cells however there was no significant change in the expression of Vimentin between T1 and T2 cells (**Supplementary Figure 5A**).

The molecular mechanism of Macc1 has been extensively investigated in colorectal cancer. Macc1 is the transcriptional activator of MET and triggers the downstream PI3K-AKT and MEK/ERK signaling pathway (53). We are currently in the process of investigating the molecular pathways perturbed in the TNBC on treatment with Lovastatin that is outside the purview of the current manuscript. Our work also underscores the critical need to target different pools of tumor cells in order to get long term clinical benefit. As proof of principle for a therapeutic approach to target the aggressive T1 tumor cells, we identified Macc1 as a key gene over expressed in T1 cells and involved in the proliferative phenotype. We went on to test the therapeutic relevance of Lovastatin, as a potential candidate for treatment of TNBC patients. Lovastatin belongs to a class of a cholesterol lowering drugs called “Statins” that are widely used in treating chronic cardiovascular conditions like hypercholesterolemia (54). Lovastatin targets the rate-limiting step of the biosynthetic mevalonate pathway by inhibiting HMG-CoA reductase, thereby lowering the cholesterol levels (51). In addition to Lovastatin, several other statins including Fulvastatin, Atorvastatin, Simvastatin and Pitavastatin have also been found to reduce Macc1 expression in multiple cancer cell lines including colorectal, gastric and pancreatic cancer (55). Crucial to our hypothesis, we found that Lovastatin did cause a significant decrease in the viability of the T1 tumor cells. This is an important step in repurposing a class of drugs to target different populations in a cancer by targeting molecules critical to different cell types.

Targeting a single subpopulation of tumor cells may not be sufficient to debulk the entire tumor. Interestingly, the 4T1GFP parental cells displayed similar proliferation and tumor growth kinetics as that of the T1 cells. The correlation heatmap of the transcriptomic analysis showed that the gene expression of 4T1GFP parental cells is similar to that of T1 cells (**Supplementary Figures 6A, B, C**). In contrast, the 4T1GFP parental cells showed significantly lower sensitivity to Lovastatin treatment compared to T1 cells (IC50 10.96 µM and 5.551 µM respectively) (**Supplementary Figure 6D**) pointing to the existence of different cell populations contributing to heterogeneous therapeutic response. In addition, the different cell types with in the tumor can engage in cooperation and competition which could further influence the response to therapy (56). It will be important to understand how these cells interact with each other and collectively impact the tumor growth and therapeutic response in future work.

Our work thus adds to the growing understanding of the contribution of different cell types to tumor growth and drug response. The ability to identify and then effectively eliminate these distinct populations within a tumor will be key to achieve long term, sustained therapeutic response and better patient prognosis in TNBC.

## Data availability statement

The datasets presented in this study can be found in online repositories. The names of the repository/repositories and accession number(s) can be found in the article/**Supplementary Material**. The datasets generated in this study can be founded under the GEO accession number GSE195995.

## Ethics statement

All animal experiments were carried out according to the regulations of the Institutional Animal Ethics Committee (IAEC), RGCB.

## Author contributions

Conceptualization, RN and AT; methodology, AT, SR, RM, PC, NK, BV, VJ, MJ, SR, AS, and RN; validation, AT; formal analysis, AT, SRT, RM, PC, NK, BV, VJ, and SR; investigation, RN; resources, RN; writing—original draft preparation, AT and RN; writing—review and editing, all authors; visualization, AT and NK; supervision, RN; project administration, RN; funding acquisition, MJ and RN. All authors contributed to the article and approved the submitted version.
