# Supplementary material for: Phenotypic heterogeneity drives differential disease outcome in a mouse model of triple negative breast cancer
Source: Front Oncol. 2023 Sep 29;13:1230647. doi: 10.3389/fonc.2023.1230647 (PMC10570535; doi:10.3389/fonc.2023.1230647)
Supplement: Supplementary file 1 [file DataSheet_1.docx]

**Supplementary information**

**
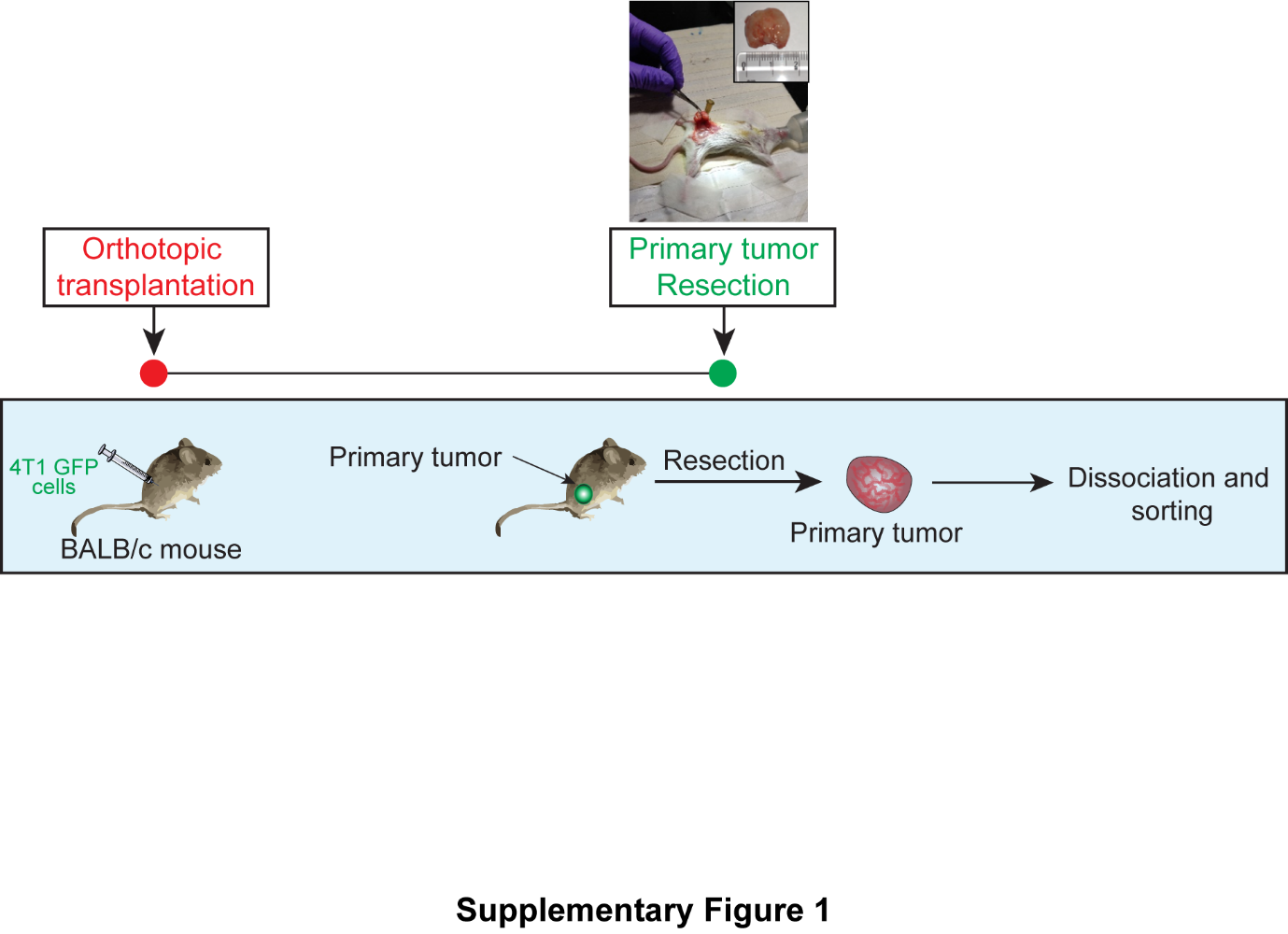
**

**Figure S1.** **Schematic showing the 4T1GFP tumor model** GFP tagged 4T1 cells were orthotopically injected into Balb/c mice. The 4T1GFP tumor thus generated was surgically resected one-month post-surgery which is then dissociated and sorted. Inset shows the primary tumor.

**
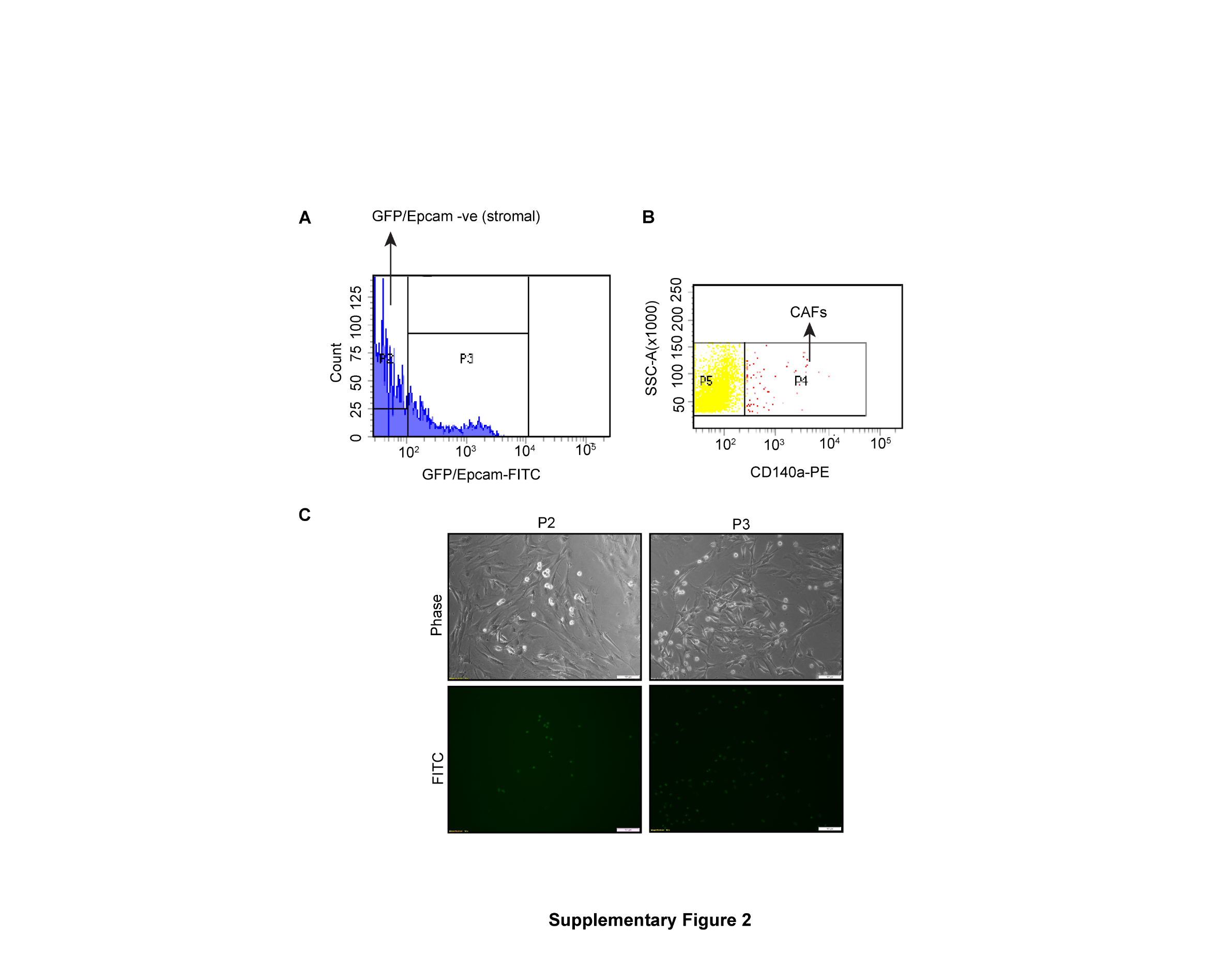
**

**Figure S2.** **Tumor cells outgrew the CAFs** (A) Histogram showing the gating of dissociated primary tumor cells based on GFP, EpCam using FACS. (B) Scatter plot showing the sorting of CAFs from the GFP -ve population based on CD140a expression. (C) Phase contrast and fluorescent microscopy images showing the CAF cell culture in P2 and P3 passages. GFP +ve tumor cells outgrew the CAF cells by the third passage (Magnification 10x).


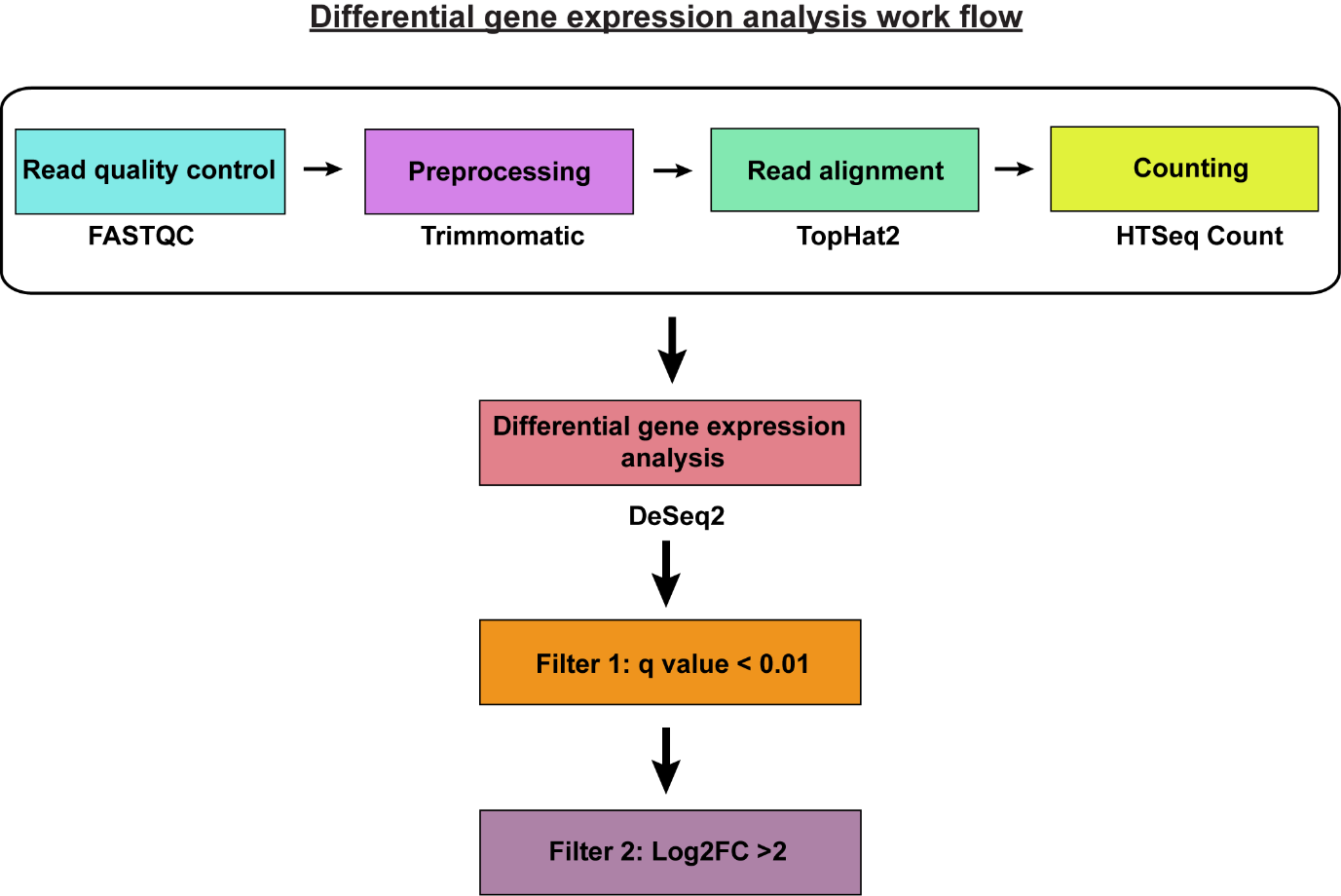


**Figure S3. Differential gene expression analysis.** Schematic showing the computational tools and filters applied for the identification of genes differentially expressed in the T1 cells that contribute to their aggressive phenotype when compared to the T2 cells.


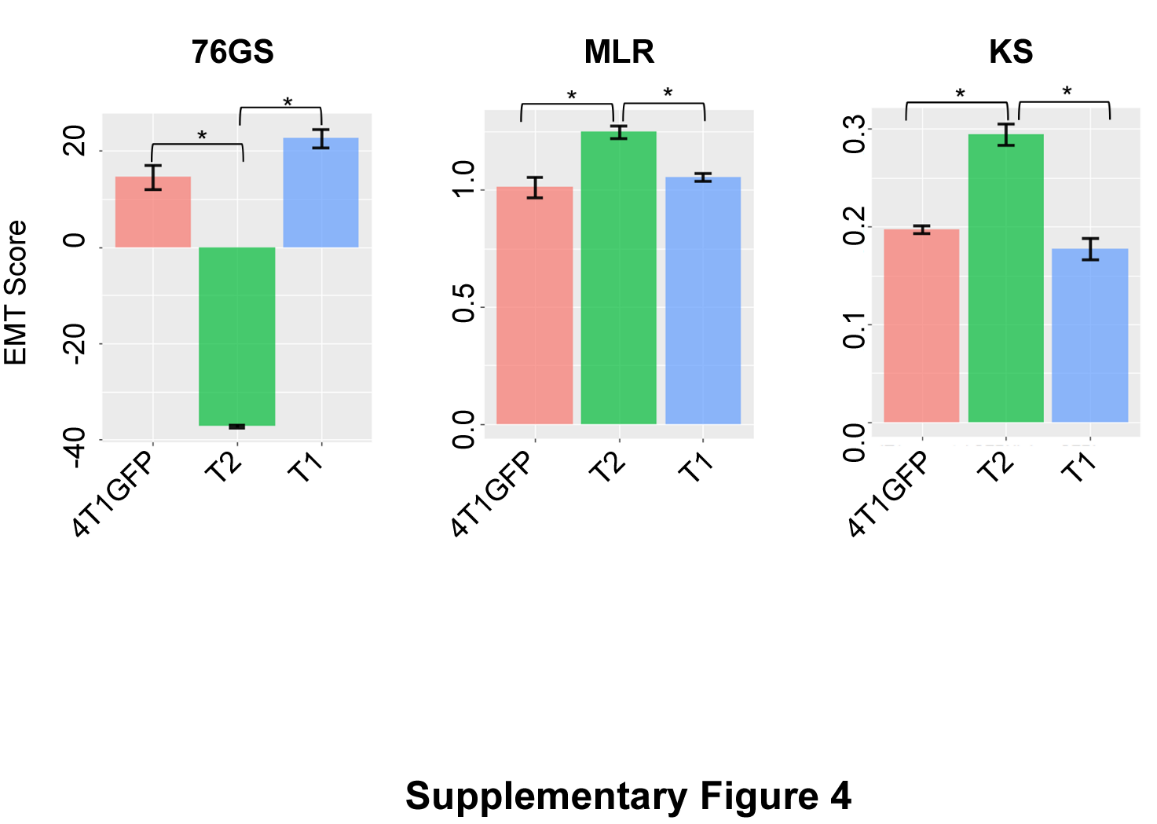


**Figure S4. EMT score analysis reveals the mesenchymal nature of T2 cells.** EMT score was calculated based on the global expression data of T1, T2 and 4T1GFP parental population using three different methods: KS, 76GS and MLR methods as described earlier (Chakraborty et al., 2020). T2 cells displayed higher mesenchymal nature in all the three methods compared to the T1 as well 4T1GFP populations.


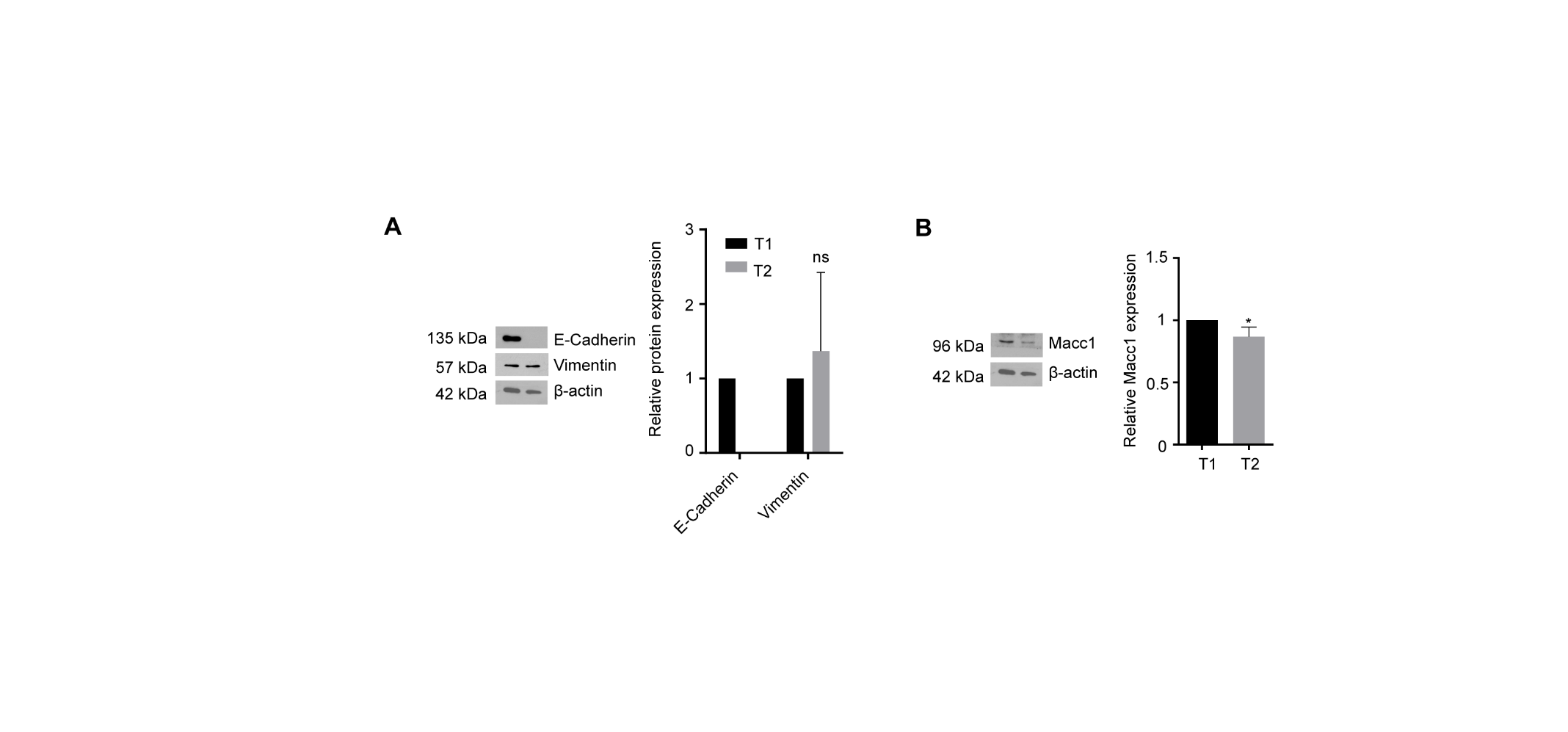


**Figure S5. Expression of E-Cadherin, Vimentin and Macc1 in T1 and T2 cells.**  Protein-level expression of E-Cadherin, Vimentin (A) and Macc1 (B) in T1 and T2 cells analyzed using western blotting. β-actin was used as loading control. Data are expressed as means ± SD, n=3, * p <0.05.


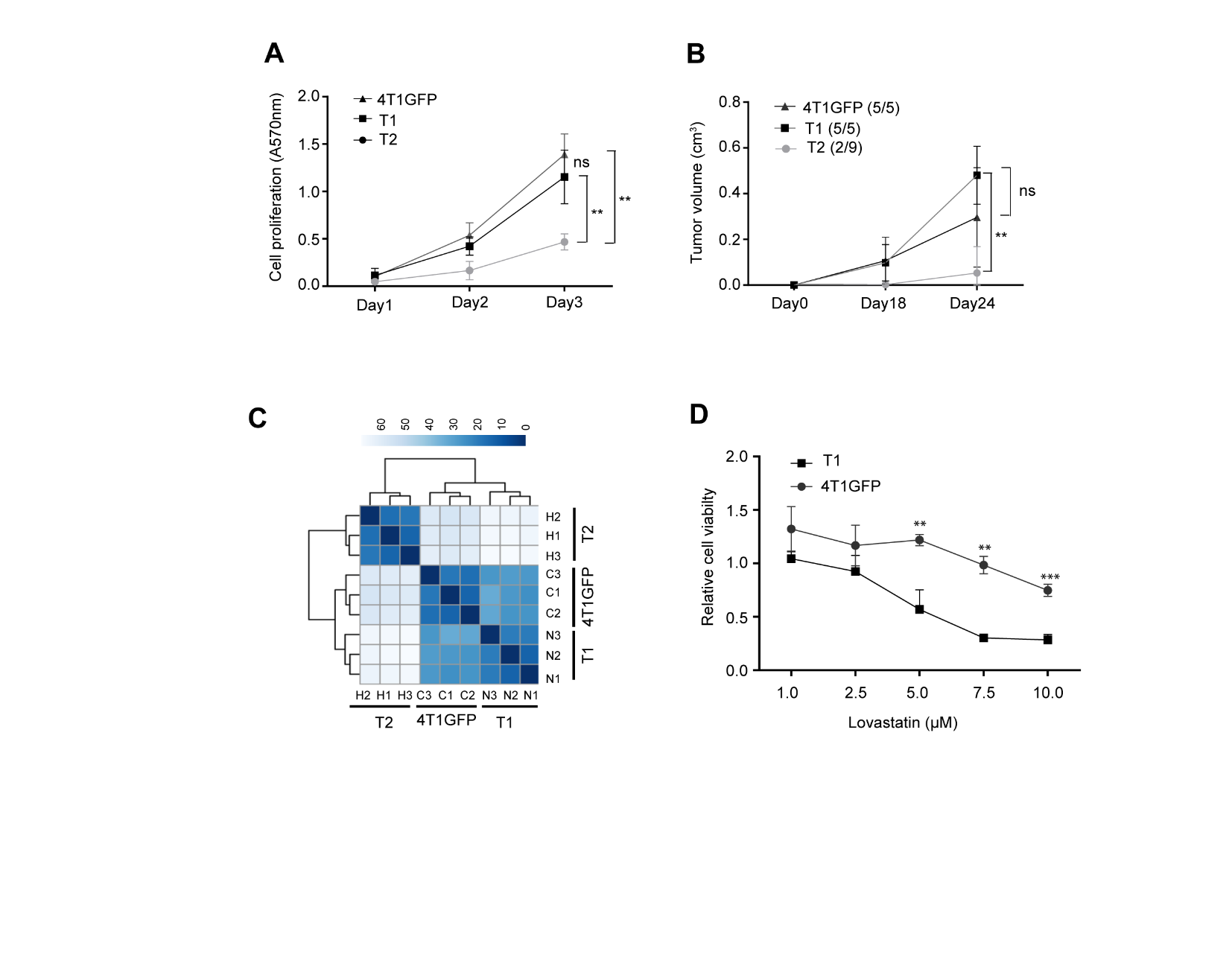


**Figure S6. Comparison between 4T1GFP and T1 cells.** A) Proliferation kinetics of 4T1GFP, T1 and T2 cells analysed using MTT assay. (B) Comparison of the tumor growth curves of mice injected with 4T1GFP (n=5), T1 (n=5) and T2 cells (n=9). In vivo tumorigenic potential of 4T1GFP, T1 and T2 tumor cells were analyzed by injecting 7x10^3^ tumor cells orthotopically into the fourth mammary fat-pad of BALB/c mice. (C) Correlation heatmap showing the gene expression of 4T1GFP parental (C1, C2, C3), T1 (N1, N2, N3) and T2 (H1, H2, H3) samples. (D) Relative cell viability of 4T1GFP and T1 cells treated with increasing concentrations of Lovastatin. 4T1GFP and T1 cells were treated with different concentrations of Lovastatin (1, 2.5, 5, 7.5 and 10 µM) and cell viability after 48 hours was measured using MTT assay. Data are expressed as means ± SD, n=3, ** p < 0.01, *** p < 0.001.

| **Mice Number** | **Number of lung metastases** |
| --- | --- |
| 1 | 7 |
| 2 | 9 |
| 3 | 0 |
| 4 | 2 |
| 5 | 14 |

**Table S1. Number of lung metastases formed by mice injected with T2 cells.** 5 mice were injected with 5x10^5^ T1 and T2 cells via tail vein to measure the metastatic potential of the cells. Lungs were harvested and metastases were visualized using hematoxylin and eosin (H&E) staining.

| **Phenotype** | **T1** | **T2** |
| --- | --- | --- |
| Morphology | Adherent | Round |
| Proliferation | High | Low |
| Self-renewal | Low | High |
| Epithelial/Mesenchymal | Epithelial | Mesenchymal |
| Disease Outcome | Aggressive | Less aggressive |

**Table S2.** **Phenotypic characterization of the heterogeneous tumor cell populations.** Table showing the comparison of the *in vitro* and *in vivo* phenotypes of the T1 cells and T2 cells obtained from the 4T1 primary tumor.

| **Gene symbol** | **Log2 FC** | **P value** |
| --- | --- | --- |
| *Fgfbp1* | 9.613072189 | 1.05E-192 |
| *Dsg2* | 9.138150775 | 1.03E-161 |
| *Dsp* | 9.121914694 | 4.26E-226 |
| *Krt14* | 8.999645887 | 1.74E-207 |
| *Cldn4* | 8.203214657 | 1.32E-113 |
| *Krt17* | 8.186290528 | 9.47E-94 |
| *Krt19* | 8.158740817 | 1.54E-116 |
| *Moxd1* | 7.782371814 | 2.49E-108 |
| *Wnt7a* | 7.673772849 | 1.56E-161 |
| *Macc1* | 7.571940264 | 5.16E-184 |
| *Ntn1* | 7.433831168 | 7.94E-84 |
| *Lncenc1* | 7.378757975 | 6.76E-146 |
| *Esrp1* | 7.355492075 | 1.05E-94 |
| *Fam83a* | 7.332464375 | 6.30E-82 |
| *Serpinb5* | 7.236955868 | 1.49E-92 |
| *Unc13b* | 7.007964245 | 4.49E-96 |
| *Anxa8* | 7.003904283 | 6.29E-110 |
| *Wisp1* | 6.968642533 | 2.77E-232 |
| *B4galnt3* | 6.841436618 | 8.31E-98 |
| *Tacstd2* | 6.815237014 | 1.08E-119 |
| *Adamts5* | 6.687909001 | 1.21E-58 |
| *C3* | 6.663375268 | 3.46E-102 |
| *Myo5b* | 6.61220331 | 1.35E-65 |
| *Tmem185b* | 6.606416729 | 2.15E-70 |
| *Mpzl2* | 6.444142475 | 2.78E-242 |
| *Ctsw* | 6.378251279 | 1.82E-79 |
| *Epcam* | 6.288581562 | 1.13E-279 |
| *Tubb2b* | 6.146066228 | 2.47E-54 |
| *Ctgf* | 6.125384483 | 0 |
| *Mal* | 6.029244829 | 2.56E-49 |
| *Pcsk6* | 6.009392893 | 4.26E-57 |
| *Mapk13* | 5.901129217 | 4.22E-185 |
| *Itgb6* | 5.867463092 | 2.24E-48 |
| *Sorbs2* | 5.860353621 | 1.64E-249 |
| *Celsr1* | 5.850815844 | 1.85E-76 |
| *Fam167a* | 5.777816816 | 1.80E-45 |
| *Edn1* | 5.758725482 | 4.87E-54 |
| *Lcp1* | 5.75021601 | 5.63E-96 |
| *Ly6e* | 5.684214796 | 2.80E-81 |
| *Cntfr* | 5.657521771 | 7.59E-41 |
| *Rasef* | 5.629302159 | 3.37E-45 |
| *Cxcl16* | 5.611714969 | 7.36E-163 |
| *Ctsc* | 5.585199733 | 1.74E-170 |
| *Piezo2* | 5.551369461 | 1.59E-45 |
| *Lama3* | 5.530057633 | 7.15E-132 |
| *Myo5c* | 5.51074033 | 7.67E-43 |
| *Postn* | 5.509196229 | 1.74E-37 |
| *Inhba* | 5.495592626 | 0 |
| *Nbl1* | 5.460232808 | 4.43E-56 |
| *AU018091* | 5.450749912 | 8.12E-61 |

**Table S3.** **50 candidate genes upregulated in the T1 compared to T2 cells** The DEGs obtained after DeSeq2 analysis was filtered based on q value < 0.01 and Log2 FC >2. The top 50 genes upregulated in the T1 population compared to T2 cells were identified and could be mediating the aggressive phenotype of T1 cells.

| **Sample names** | **Sample** | **76GS** | **MLR** | **KS** |
| --- | --- | --- | --- | --- |
| N3 | T1 | 24.8464719 | 1.0381426 | 0.1736343 |
| N2 |  | 21.9802142 | 1.0704948 | 0.190078 |
| N1 |  | 21.0816129 | 1.062039 | 0.1683389 |
| H1 | T2 | -37.450694 | 1.2640291 | 0.2934783 |
| H2 |  | -36.86977 | 1.2184623 | 0.3062988 |
| H3 |  | -37.275363 | 1.2664043 | 0.2845596 |
| C1 | 4T1GFP(Parental) | 11.9937033 | 1.0375892 | 0.2017837 |
| C3 |  | 17.1188572 | 0.9607885 | 0.1939799 |
| C2 |  | 14.5749679 | 1.0378739 | 0.1953735 |

**Table S4. EMT scores for T1, T2 and 4T1GFP cells** EMT scores were calculated using three different methods (KS, 76 GS and MLR methods) for T1, T2 and 4T1GFP (parental) cells.

| **Name** | **Catalogue number** | **Concentration used** |
| --- | --- | --- |
| Macc1 | 5197 | 1:1000 |
| E-Cadherin | 2E410 | 1:1000 |
| Vimentin | 5741 | 1:2000 |
| β-actin | A-5441 | 1:50000 |

**Table S5. Antibodies used for western blotting**

**References**

CHAKRABORTY, P., GEORGE, J. T., TRIPATHI, S., LEVINE, H. & JOLLY, M. K. 2020. Comparative Study of Transcriptomics-Based Scoring Metrics for the Epithelial-Hybrid-Mesenchymal Spectrum. *Front Bioeng Biotechnol,* 8**,** 220.
